# Elimination of Iron Deficiency Anemia and Soil Transmitted Helminth Infection: Evidence from a Fifty-four Month Iron-Folic Acid and De-worming Program

**DOI:** 10.1371/journal.pntd.0002146

**Published:** 2013-04-11

**Authors:** Gerard J. Casey, Antonio Montresor, Luca T. Cavalli-Sforza, Hoang Thu, Luong B. Phu, Ta T. Tinh, Nong T. Tien, Tran Q. Phuc, Beverley-Ann Biggs

**Affiliations:** 1 Department of Medicine, The University of Melbourne, The Royal Melbourne Hospital, Parkville, Australia; 2 World Health Organization, Geneva, Switzerland; 3 World Health Organization, Western Pacific Regional Office, Manila, Philippines; 4 Centre of Malariology, Parasitology and Entomology, Yen Bai, Vietnam; 5 National Institute of Malariology, Parasitology and Entomology, Hanoi, Vietnam; 6 Victorian Infectious Diseases Service, The Royal Melbourne Hospital, Parkville, Australia; University of California Los Angeles, United States of America

## Abstract

**Background:**

Intermittent iron-folic acid supplementation and regular de-worming are effective initiatives to reduce anemia, iron deficiency, iron deficiency anemia, and soil transmitted helminth infections in women of reproductive age. However, few studies have assessed the long-term effectiveness of population-based interventions delivered in resource-constrained settings.

**Methodology/Principal Findings:**

The objectives were to evaluate the impact of weekly iron-folic acid supplementation and de-worming on mean hemoglobin and the prevalence of anaemia, iron deficiency, and soil transmitted helminth infection in a rural population of women in northern Vietnam and to identify predictive factors for hematological outcomes. A prospective cohort design was used to evaluate a population-based supplementation and deworming program over 54 months. The 389 participants were enrolled just prior to commencement of the intervention. After 54 months 76% (95% CI [68%, 84%]) were taking the iron-folic acid supplement and 95% (95% CI [93%, 98%]) had taken the most recently distributed deworming treatment. Mean hemoglobin rose from 122 g/L (95% CI [120, 124]) to 131 g/L (95% CI [128, 134]) and anemia prevalence fell from 38% (95% CI [31%, 45%]) to 18% (95% CI [12%, 23%]); however, results differed significantly between ethnic groups. Iron deficiency fell from 23% (95% CI [17%, 29%]) to 8% (95% CI [4%, 12%]), while the prevalence of iron deficiency anemia was reduced to 4% (95% CI [1%, 7%]). The prevalence of hookworm infection was reduced from 76% (95% CI [68%, 83%]) to 11% (95% CI [5%, 18%]). The level of moderate or heavy infestation of any soil-transmitted helminth was reduced to less than 1%.

**Conclusions/Significance:**

Population-based interventions can efficiently and effectively reduce anemia and practically eliminate iron deficiency anemia and moderate to heavy soil transmitted helminth infections, maintaining them below the level of public health concern.

## Author Summary

Nutritional deficiencies are common in women in rural and disadvantaged regions, and, in particular, iron and folate deficiencies may impact their health and that of their offspring. Weekly iron-folic acid supplementation and regular deworming help to prevent these deficiencies, yet many women in developing countries do not have access to these interventions. Using the local health system, we implemented a weekly iron-folic acid supplementation program with regular deworming for approximately 250,000 women in a rural mountainous province of Viet Nam. We present the results of a survey of women's iron and worm infection status after the program has been running for 54 months. Overall, anemia prevalence fell from 38% to 18%, iron deficiency fell from 23% to 8% and the prevalence of iron deficiency anemia was reduced to 4%. The level of moderate or heavy infestation of any soil-transmitted worm infection was reduced to less than 1%. These results show that a health system-based program can reduce and maintain the prevalence of these diseases in the community at a level where they do not constitute a public health risk.

## Introduction

Iron deficiency is one of the most common nutritional deficiencies affecting women of reproductive age and children [1]. The increased demand for iron in these groups is often exacerbated by limited intake of heme iron, and hookworm infection with associated gastrointestinal blood loss. Globally, 42% of pregnant women are anemic with iron deficiency being the most common cause [2]. Maternal iron deficiency anemia is associated with intra-uterine growth retardation, low birth weight, preterm birth, and increased risk of maternal and infant mortality [3]. Recent evidence suggests that women who are iron sufficient at conception and in early pregnancy have improved neonatal outcomes [4], [5]. The current recommended strategy for the prevention of iron deficiency in pregnancy is daily iron-folic acid supplementation, taken from the first antenatal visit until three months post-partum [6]. As a population-based strategy, this approach has generally been ineffective due to variable coverage and poor compliance [7], [8].

There are also major benefits for women to be iron sufficient when not pregnant, in particular, improved resistance to infection and greater work capacity and productivity [3], [9]. In rural communities where women are significant contributors to household food production, these are important considerations and there is a growing awareness that micronutrient deficiencies, and iron deficiency in particular, carry a major economic cost [10], [11], [12]. WHO now recommends that where the prevalence of anemia in women of reproductive age is greater than 20%, supplementation with a weekly dose of iron-folic acid should be considered as a strategy for the prevention of iron deficiency [13], [14].

Where dietary intake of heme iron is inadequate, regular iron-folic acid supplementation needs to be maintained over extended periods to avoid loss of iron stores. Modeling studies on iron absorption and loss by Lynch (2000) suggest that non-pregnant adolescents revert to their pre-supplementation levels of iron five months after stopping a weekly 60 mg supplementation regimen [15]. The challenge is to develop strategies for the promotion, implementation and sustained funding of effective community-based iron supplementation programs for women of reproductive age.

In November 2005, we undertook a survey of 389 women aged 16–45 years in two districts of a northern rural province in Vietnam, and found that the rates of anemia, iron deficiency, iron deficiency anemia and hookworm infection were 38%, 23%, 18% and 76% respectively [16]. After an initial pilot project of regular de-worming and weekly iron-folic acid supplementation [17], the provincial health services took control of and expanded the program to all districts in the province [18]. Population impact surveys showing improvements in all indicators over 30 months have been previously reported [17], [19], [20].

In the current study we evaluated hematological and parasitological outcomes, and compliance in a prospective cohort of women who were monitored from program inception. The objectives were to evaluate the impact of weekly iron-folic acid supplementation and de-worming on mean hemoglobin and the prevalence of anaemia, iron deficiency and soil transmitted helminth infection in a rural population of women in northern Vietnam, and to identify predictive factors for hematological outcomes.

## Methods

### Ethics

Extensive consultation was undertaken between the project team, communities and community leaders, as well as liaison with village, district and provincial health staff. Village health workers provided participants with information regarding the surveys and written informed consent was documented at the time of enrolment in the surveys. The project was approved by the Human Research Ethics Committees of the National Institute of Malariology, Parasitology and Entomology (Hanoi, Vietnam), the Walter and Eliza Hall Institute of Medical Research (Melbourne, Australia) and Melbourne Health (Melbourne, Australia).

### Study Rationale and Design

This study was a collaboration between the University of Melbourne, the National Institute of Malariology, Parasitology and Entomology, Vietnam, and the Province of Yen Bai. . A key element of the design was the demonstration of a population-based intervention for the benefit of women of reproductive age, which could later be expanded to other provinces. Provincial authorities, community leaders and researchers felt strongly that it would be unethical to deny a section of the eligible population treatment for helminth infections over the course of the project. Therefore, a prospective cohort study with serial follow up surveys was identified as the most appropriate design for this situation.

Haemoglobin, serum ferritin, and soil transmitted helminth infection were the clinical parameters assessed at baseline and each follow-up survey. Questionnaires were designed to cover as many foreseeable confounders as possible: age, ethnicity, marital status, number of children, occupation, attained education level, frequency of meat consumption, wearing of footwear while working, type of latrine used and frequency and timing of hand washing.

### Study Location and Population

Vietnam has 54 identified ethnic groups of which Kinh is the predominant (86%) [21]. Yen Bai province is one of the 63 provinces in Vietnam. It is a mountainous region 180 km north-west of Hanoi with a largely rural economy, widespread poverty and diverse ethnic groups. The total population is approximately 735,000 [22]. The districts of Tran Yen and Yen Binh each have roughly 26,000 women aged between 15 and 45 years. Approximately 65% are Kinh while Tay and Dao minorities make up a further 26% (Dr. Luong Ba Phu, personal communication).

### Intervention

In May 2006, a pilot project providing weekly iron-folic acid together with regular de-worming for all women of reproductive age was initiated in two districts of Yen Bai province, covering approximately 50,000 women aged between 15 and 45 years. Prior to commencement, two staff of each district Department of Preventive Medicine, two nurses from each commune health station and all village health workers in the two districts (a total of 680 health personnel) were trained about the causes, health risks, treatment and prevention of anemia and hookworm infection and received promotional and educational materials for use in their communities and training on their effective usage. The educational materials included advice on wearing footwear in the fields while working. The intervention consisted of one iron-folic acid tablet taken weekly (200 mg ferrous sulphate equivalent to 60 mg elemental iron and 0.4 mg folic acid, UNICEF, Copenhagen), and one albendazole tablet (400 mg, UNICEF, Copenhagen) taken every four months for the first year. At the end of the first year, the de-worming schedule was changed to 6-monthly in the light of the observed significant reduction in worm burden during the first twelve months. Iron-folic acid tablets were sourced in blister packs from Nam Ha Pharmaceutical Joint Stock Company, a Vietnamese pharmaceutical manufacturer. We were unable to obtain supplements that contained the higher folic acid levels recommended for intermittent dosing, so we used the available combination supplements provided by UNICEF and Nam Ha Pharmaceutical Company for use in pregnant women. Pregnant women were not provided deworming treatment at any time during the intervention as this is proscribed during pregnancy by the Vietnamese Ministry of Health.

The Provincial Preventive Medicine Department supervised the distribution of iron-folic acid and albendazole tablets to district and commune levels of the health service. Albendazole tablets were administered as observed treatment on locally designated days either at the commune health station or supervised in the village by a commune health worker. Village health workers distributed iron-folic acid tablets to women on a monthly basis. The processes undertaken to initiate, promote, and maintain the intervention are described elsewhere [18].

The positive results of an evaluation survey after 12 months [17] led to program expansion in May 2008 to target all women of reproductive age in the province (approximately 250,000 women). Direct management of the program was taken over by the provincial health authorities. Assistance was provided through the National Institute of Malariology, Parasitology and Entomology, including provision of educational and promotional materials, training of health care staff throughout the province, and advocacy at national and international levels. Funding for the expansion was provided through the initial project grant (Atlantic Philanthropies Inc. 2006–2009), the World Health Organization Western Pacific Regional Office (2010–2011) and Irish Aid (2012).

### Selection of Participants

A sample size of 350 was calculated as sufficient to detect a 10% change in arithmetic mean hemoglobin with a power of 90% (α = 0.05). Allowing for no-shows, 33 villages and 13 women per village were selected using a population proportional to size clustering method. In total, the cohort consisted of 389 women who participated in the baseline survey, 354 of whom provided a venous blood sample.

### Monitoring and Evaluation

Haematological and parasitological outcomes were evaluated in the cohort at 3, 12, 30 and 54 months after commencement of the intervention. Respondents completed a structured interview, which included questions about their compliance with the intervention, and provided blood and fecal samples.

At each follow-up survey, women were invited to access and discuss their current hemoglobin and soil-transmitted helminth results in light of their previous results and compliance with the weekly iron-folic acid and de-worming program. Women who were identified as severely anemic (hemoglobin<70 g/L) were referred to the commune health station.

### Samples and Testing

Sample collection and laboratory analysis has been previously reported [16] and was the same in each of the surveys. In brief, trained phlebotomists took a 3 ml venous blood sample, one drop of which was used for hemoglobin analysis (Hemocue 201+, Hemocue AB, Angleholm, Sweden) and the remainder centrifuged at 1200 g for 10 minutes and the serum aliquots placed on wet ice prior to storage at −20°C at the end of each day. Serum ferritin was measured using a sandwich immunoenzymatic assay (Beckman Coulter Access Reagents, Fullerton, CA). Anemia was defined as a hemoglobin concentration of <120 g/L in accordance with WHO recommendations for women of reproductive age [6]. The laboratory serum ferritin reference range for adult females was 15 µg/L-200 µg/L. Iron deficiency was defined as serum ferritin<15 µg/L. A cut-off of 200 µg/L was used to identify high serum ferritin levels (current chelator-prescribing guidelines recommend no treatment be given for ferritin levels below 500 µg/L) [23]. Laboratory analyses were conducted at South Eastern Area Laboratory Services, Sydney, Australia (now South Eastern Sydney and Illawarra Area Health Service).

Faecal sample analysis for soil transmitted helminth eggs was conducted at the field site using standard Kato-Katz methodology [24]. Classification of hookworm infection was based on WHO guidelines of 0 eggs per gram (epg) as no infection, >0 and <2000 epg as mild infection and >2000 epg as moderate or heavy infection. Soil transmitted helminth infection was categorized as positive if eggs of hookworm (*Ancylostoma duodenale* and *Necator americanus*), *Ascaris lumbricoides* or *Trichuris trichiura* were detected in the stool sample.

### Statistical Analysis

Primary outcomes were mean hemoglobin and serum ferritin and changes in prevalence of anemia, iron deficiency, iron deficiency anemia and soil transmitted helminth infection. Data were assembled as longitudinal survey panel data for analysis. For continuous variables the mean at each time point was calculated; arithmetic mean for hemoglobin and geometric mean for serum ferritin.

Changes in means and prevalences of hematological parameters over time were analysed on the entire cohort to assess the overall impact of the intervention. Means were calculated incorporating village clustering to provide adjusted confidence intervals.

Regression analyses were conducted to assess factors potentially affecting the impact of iron supplementation and deworming treatment on hematological outcomes in women who reported taking supplements. These used a multilevel mixed-effects model accounting for both village clustering and repeated observations of individuals to produce adjusted confidence intervals. The linear regression model tested continuous variables and incorporated: age, marital status, number of children, hookworm, Ascaris and Trichuris worm burden, occupation, years of education, number of meat meals per week and ethnic group. The logistic regression model tested binary and categorical variables and incorporated: age less than 35 years or 35 years and older, married or unmarried/widowed, agricultural or non-agricultural employment, infection with hookworm, Ascaris or Trichuris, eating more than 3 meat meals per week or not, up to 9 years of education (the average) or more, having 0, 1 or 2, or more than 2 children and Kinh or non-Kinh ethnicity. Due to the habit of working outdoors in bare feet, a binary classification of indoor/outdoor work was used as a proxy for exposure risk to soil transmitted helminth infection.

Statistical analysis was performed using Stata Revision 11 (StataCorp, 2009, College Station, Texas).

## Results

**Figure 1:**
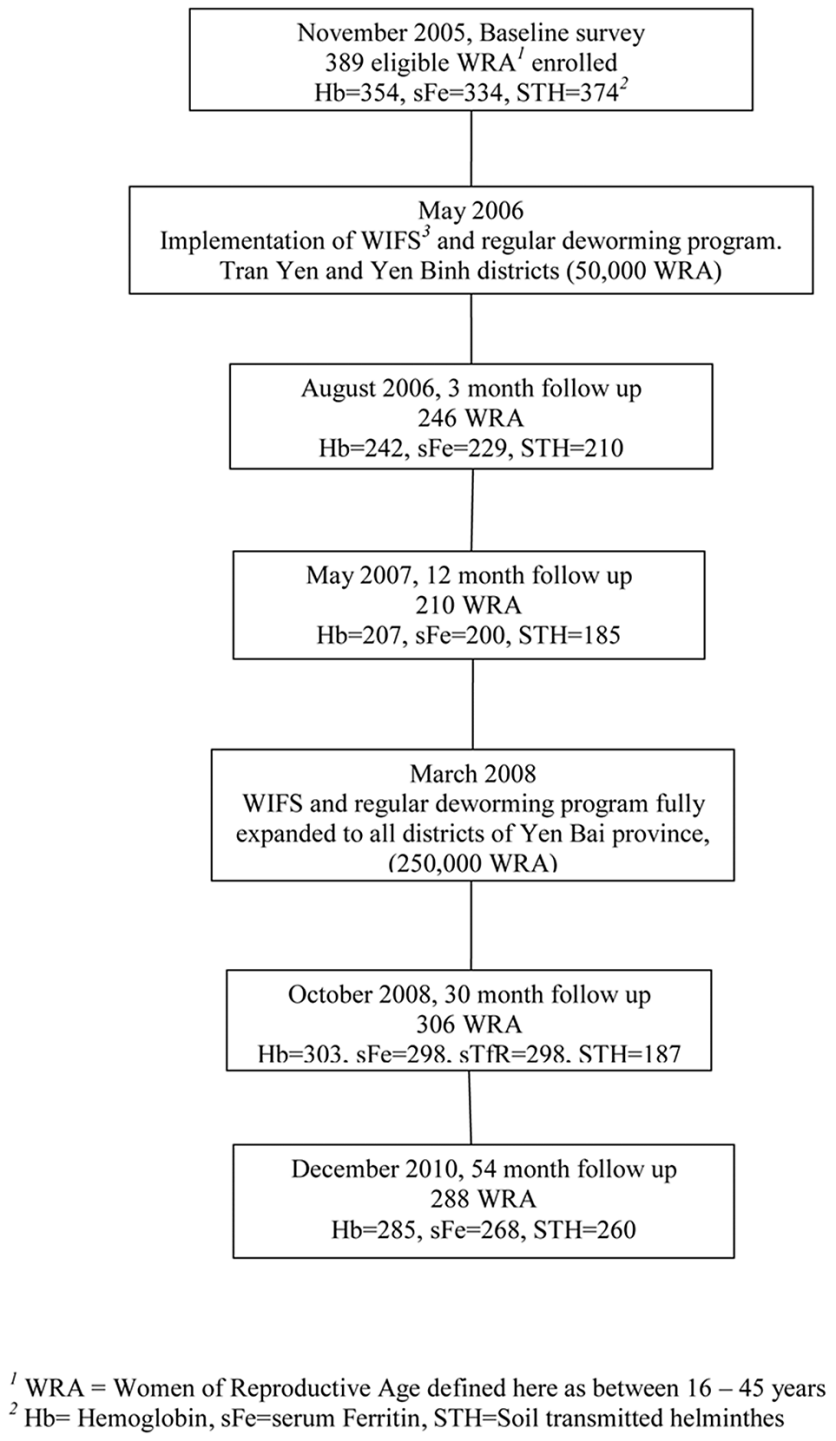
Timeline of surveys and intervention. *^1^* WRA = Women of Reproductive Age defined here as between 16–45 years. *^2^* Hb = Hemoglobin, sFe = serum Ferritin, STH = Soil transmitted helminthes. *^3^* WIFS = Weekly Iron Folic acid Supplementation.

**Figure 2:**
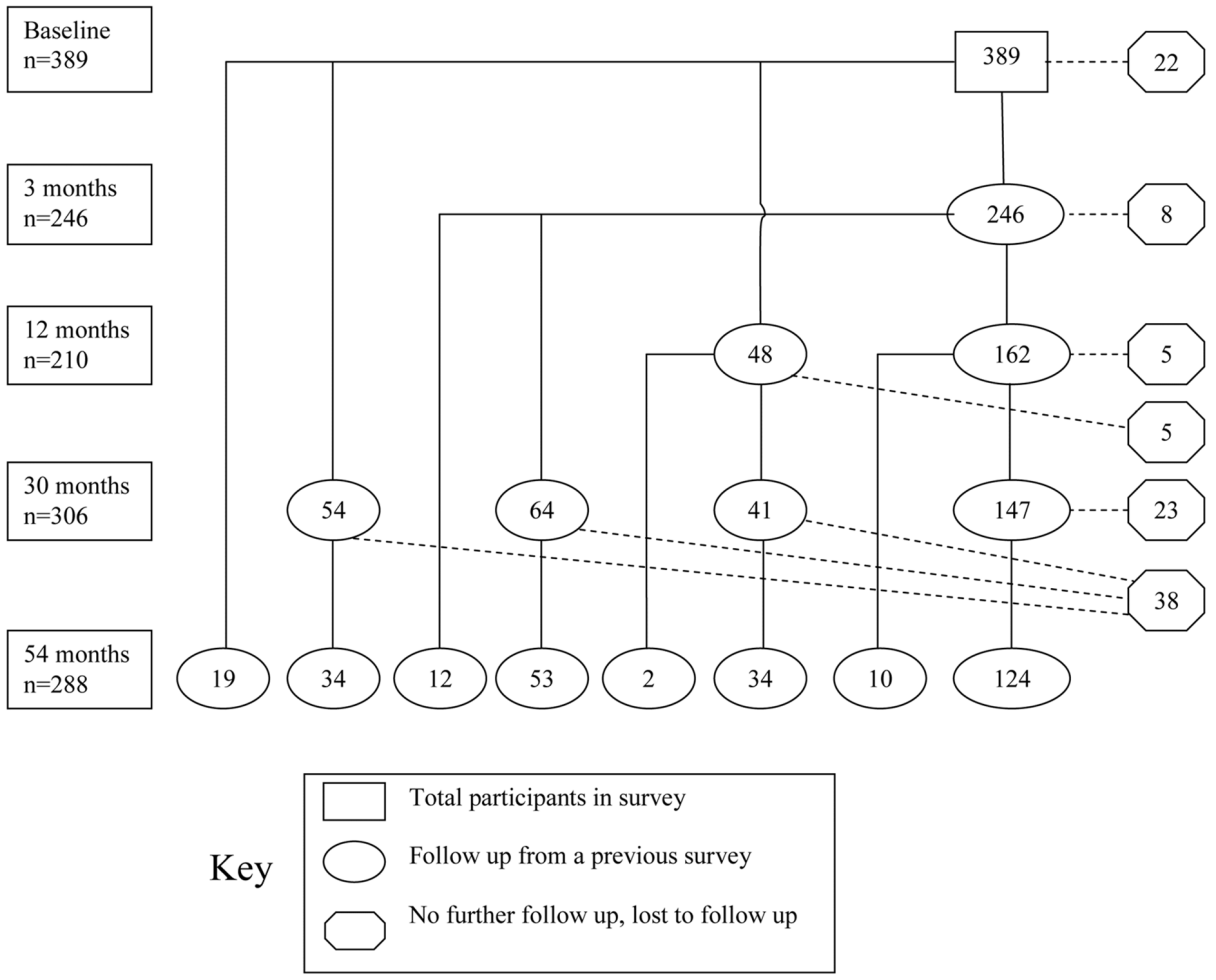
Flowchart of participant follow up. Those lost to follow up did not re-present at any later survey. Of the 389 baseline cohort women, 288 (74% and the sum of the ovals horizontal from the 54 month survey rectangle) returned for the 54-month survey of whom 124 attended every survey as shown by the direct connecting line from Baseline to the 54 month follow-up. The other 164 women who attended the 54 month survey missed one or more intermediate surveys but were contacted and followed up subsequently. For example, 48 women who attended the Baseline survey missed the 3 month survey but did attend at 12 months. Of these, 41 attended at 30 months and 34 of them attended at 54 months. Another two women from the 12 month survey missed the 30 month survey but attended at 54 months.

The timing of program expansion and evaluation surveys are shown in **Figure 1** and the participant flow is shown in **Figure 2** in which the rectangular boxes at the left indicate the total number of women enrolled in each survey, the ovals show the number of women followed up from previous surveys and the octogons at the right show the number of women from each group who were not part of any subsequent survey. This shows 101 women were permanently lost to follow-up over the 54 months. For the most part, local health staff advised that these women had left the area. Failure to attend a survey was due to a number of factors: working in another district or province, unwillingness to provide a blood sample and, for the 3 month survey, a serious disruption due to monsoonal rain. The number of women included in these analyses at 3 months, 12 months, 30 months and 54 months were 238, 201, 284 and 217 respectively.

At the 54 month survey, three women brought a stool sample but left immediately after registering without providing hemocue or blood sample or completing the questionnaire, 17 declined to provide a blood sample but did agree to a finger prick for hemoglobin and 28 did not provide a stool sample.

The demographic characteristics of the women at baseline and 54 months are shown in **Table 1**. The ethnic make-up of the survey participants and the predominance of two-child families remained unchanged. As expected, there was a decrease in the number of unmarried women and women without children. There was no change in the level of education attained or the proportion of women working in agriculture. There was no significant change in the number of meat meals eaten per week or egg/fish consumption over the period of the study. There was some increase in the frequency of hand-washing although this was not significant (data not shown). The percentage of women who did not wear shoes while working dropped from 12% to 2% (data not shown). While there was an overall drop in compliance with taking iron-folic acid supplements at 54 months (76%) compared to 30 months (87% [19]), the identified difference in non-compliance between Kinh and non-Kinh groups was not significant (Odds ratio 2.1 [95% CI 0.94, 4.74], *p* = 0.07). The proportion of women accessing de-worming treatment increased from 88% at 30 months to 95% at 54 months (data not shown).

**Table 1:** Demographic information for a cohort of 389 women with access to a deworming and weekly iron-folic acid program at baseline and 54 months post-implementation.

		Baseline	54 month survey
		Mean (+SD)/Freq (%)	Mean (+SD)/Freq (%)
Age		30.1 (+8.0)	36.2 (+7.7)
Meat meals per week		4.1 (+2.9)	4.2 (+3.0)
Marital status	Married	332 (85%)	273 (96%)
	Single	54 (14%)	7 (2%)
	Widowed/divorced	3 (1%)	4 (1%)
Number of children	None	65 (17%)	10 (3%)
	1	85 (22%)	40 (14%)
	2	171 (44%)	171 (60%)
	3	46 (12%)	45 (16%)
	More than 3	22 (6%)	20 (7%)
Occupation	Agriculture	333 (86%)	251 (87%)
	Other	55 (14%)	37 (13%)
Education	6 years or less	87 (22%)	78 (27%)
	7–9 years	217 (56%)	153 (54%)
	10–12 years	73 (19%)	43 (15%)
	>12 years	11 (3%)	11 (4%)
Ethnicity	Kinh	259 (67%)	183 (64%)
	Tay	46 (12%)	41 (14%)
	Cao Lan	28 (7%)	18 (7%)
	Dao	51 (13%)	41 (14%)
	Other	4 (1%)	5 (2%)
Took last de-worming treatment*	Yes		275 (95%)
	No		13 (5%)
Taking Fe+Fo supplements*	Yes		217 (76%)
	No		68 (24%)

* Women did not have access to regular de-worming treatment or free iron-folic acid supplements prior to the intervention.

The change in outcome measures for the survey population over time is shown in **Table 2**. At baseline Kinh women were less likely to be anemic (Odds ratio 0.56 [95% CI 0.33, 0.96], *p* = 0.03) or to have iron deficiency anemia (Odds ratio 0.49 [95% CI 0.27, 0.90], *p* = 0.02) than non-Kinh women. The two groups were equally likely to be iron deficient despite Kinh women eating more meat (Odds ratio 3.2 [95% CI 1.4, 7.2], *p* = 0.006). The two groups were also equally likely to have hookworm infection.

**Table 2:** Change over time in assessed outcomes for all survey participants from baseline to 54 months post-implementation of iron-folic acid supplementation and de-worming program.

	Baseline survey			3 month survey			12 month survey			30 month survey			54 month survey		
	N	Mean	95% CI	N	Mean	95% CI	N	Mean	95% CI	N	Mean	95% CI	N	Mean	95% CI
Hemoglobin (g/L)	354	122	120 124	242	126	124 128	207	130	128 132	303	130	128 132	285	131	128 134
Ferritin (µg/L)*	334	28.0	23.9 32.7	229	37.4	32.5 43.0	200	47.6	41.9 54.2	298	52.4	45.0 60.9	268	53.8	46.5 62.3
Anemia (Total)	354	38%	31% 45%	242	26%	20% 33%	207	19%	13% 24%	303	19%	14% 24%	285	18%	12% 23%
*Kinh*	235	33%	25% 41%	153	22%	15% 28%	134	16%	10% 22%	192	15%	8% 21%	181	12%	7% 17%
*Non-Kinh*	119	47%	36% 58%	89	35%	22% 48%	73	26%	15% 37%	111	26%	17% 35%	104	29%	19% 38%
Iron deficiency	334	23%	17% 29%	229	13%	9% 17%	200	7%	4% 10%	298	9%	5% 13%	268	8%	4% 12%
*Kinh*	219	21%	13% 28%	144	12%	7% 17%	129	6%	2% 10%	189	8%	2% 14%	169	6%	2% 10%
*Non-Kinh*	115	28%	17% 38%	85	15%	7% 23%	71	8%	3% 14%	109	11%	6% 16%	99	12%	3% 21%
IDA (Total)	334	18%	13% 23%	229	10%	6% 13%	200	3%	<1% 6%	297	6%	3% 8%	268	4%	1% 7%
*Kinh*	219	14%	9% 19%	144	7%	4% 10%	129	3%	<1% 7%	188	3%	<1% 7%	167	2%	<1% 5%
*Non-Kinh*	115	25%	15% 35%	85	14%	7% 21%	71	2%	<1% 7%	109	9%	5% 14%	99	6%	<1% 12%
Hookworm	374	76%	68% 83%	210	57%	49% 65%	185	30%	21% 38%	187	22%	12% 32%	260	11%	5% 18%
*A. lumbricoides*	374	19%	13% 27%	210	7%	1% 12%	185	4%	1% 7%	187	4%	1% 7%	260	5%	<1% 9%
*T. trichiura*	374	29%	23% 35%	210	22%	14% 30%	185	11%	4% 18%	187	10%	4% 15%	260	3%	<1% 5%

* geometric mean.

IDA = iron deficiency anemia.

After 54 months, anemia prevalence stabilised at about 18% and the significant difference in prevalence between Kinh and non-Kinh women found at baseline was more evident (Odds ratio 0.32, [95% CI 0.17, 0.60], *p* = 0.001). The anemia prevalence for Kinh women fell by 70% over the period of the study compared to a reduction of 38% for non-Kinh women. Significant differences between the two groups were not observed in the prevalence of iron deficiency or iron deficiency anemia. Overall iron deficiency fell from 23% to 8%, and iron deficiency anemia from 18% to 4% after 54 months of the intervention.

**Figure 3:**
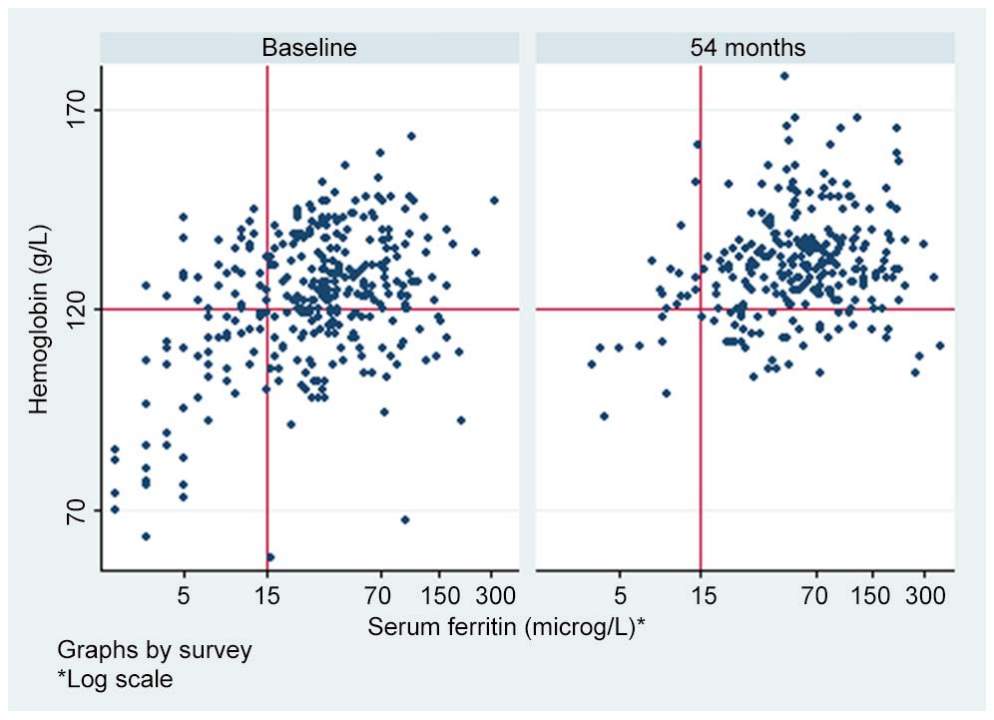
Comparative paired hemoglobin and serum ferritin values scatter graph. Vertical and horizontal lines represent lower limit of normal for ferritin and hemoglobin respectively.

After 54 months mean hemoglobin was 131 g/L, which was the same as the level recorded after 12 months of intervention. In contrast, mean ferritin levels continued to rise over the period of the intervention to a level almost double that recorded at baseline. The changes in hemoglobin and ferritin are shown in **Figure 3**.

**Figure 4:**
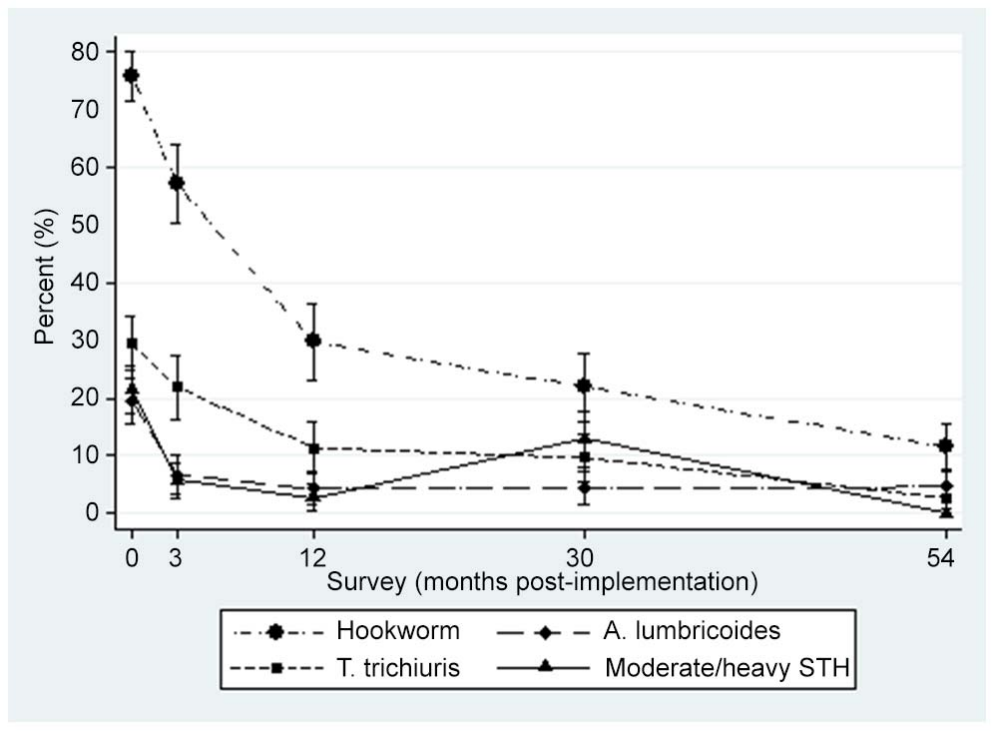
Change in prevalence over time for Hookworm, *T. trichiuris*, *A. lumbricoides*, and moderate/high intensity soil transmitted helminth infection.

The prevalence of hookworm and *T. trichiura* infections continued to fall over the 54 months of evaluation. However, the prevalence of *A. lumbricoides* remained at about 4% after the first 12 months (**Figure 4**). After 54 months, no woman had a hookworm count greater than 300 eggs/gram and the prevalence of moderate/heavy intensity infection with any soil transmitted helminth had fallen to less than 1%.

The results of regression analyses for hematological and parasitological outcomes for women who reported taking the supplements and anthelminths are shown in **Table 3**. Ethnicity was strongly associated with lower hemoglobin, anemia and iron deficiency anemia. Older age, higher meat consumption and non-agricultural employment were significantly associated with higher serum ferritin, while low meat consumption was the only significant factor for iron deficiency. Overall, non-Kinh women consumed less meat than Kinh women (coeff −0.32 [95% CI −0.50, −0.15], *p*<0.001) however this was not associated with differences in hemoglobin or anemia between the groups. Of those women who reported still taking the weekly supplement at 54 months post-intervention, Kinh women remained less likely to be anemic than non-Kinh women (Odds ratio 0.28 [95% CI 0.15, 0.55], *p*<0.001).

**Table 3:** Regression analyses for women who reported taking weekly iron-folic acid supplement.^1^

Linear regression				
Output	Variable	Coef	95% CI	*P*
Hemoglobin	Ethnicity	−1.38	−2.29 −0.47	0.003
Serum Ferritin	Age	0.02	0.01 0.08	<0.001
	Occupation	−0.27	−0.48 −0.07	0.009
	Meat (meals/week)	0.05	0.02 0.05	<0.001

Logistic regression				
Output	Variable	Odds ratio	95% CI	*P*
Anemia	Kinh	0.32	0.17 0.58	<0.001
Iron deficiency	Meat (>3 meals/week)	0.46	0.22 0.94	0.03
Iron deficiency anemia	Kinh	0.42	0.20 0.87	0.02
	Hookworm positive	2.21	1.89 4.50	0.03

1 Multilevel mixed-effects model used to account for both village clustering and repeated observations of individuals to produce adjusted confidence intervals.

Across all post-implementation surveys, a total of 37/995 (3.7%) ferritin readings were above 200 µg/L. Thirteen women had more than one high serum ferritin reading; 8 women were Kinh and 5 were of Tay ethnic background. Six of the women were 40 years of age or older. The highest recorded ferritin was 538.1 µg/L in a Dau woman who recorded 2 other readings of less than 50 µg/L. No other readings above 500 µg/L were recorded in the cohort over the period of the study. At the 54 month survey, 14/268 (5.2%) women had a serum ferritin of 200 µg/L or higher, 4 of whom also had results ≥200 µg/L at 30 months.

## Discussion

We report the hematological and parasitological outcomes of a population-based program of weekly iron-folic acid supplementation and regular deworming for women of reproductive age after 54 months of implementation in a rural mountainous province of Vietnam, and investigate the factors that influence these outcomes. Compliance with both deworming and weekly iron-folic acid supplements remained high; the population mean hemoglobin level stabilized at 131 g/L and serum ferritin maintained a steady increase. Anemia, iron deficiency and iron deficiency anemia were all significantly reduced from pre-intervention levels to a point where they would no longer be regarded as significant public health threats. Importantly, morbidity associated with soil transmitted helminthes was eliminated based on the WHO definition of less than 1% prevalence of moderate/heavy intensity infection,

Anemia as a proxy measure for population iron deficiency is unreliable [25]. A contributing factor to this is the presence of various genetic hemoglobin disorders. These can affect the capacity of studies measuring hemoglobin to accurately reflect the level of iron deficiency and the impact of iron supplementation. It is estimated that 13.2% of the population of the WHO Western Pacific region, which includes Vietnam, carry some hemoglobin variant [26]. We and others have previously reported an apparent inability of iron supplementation programs to reduce the prevalence of anemia below 18%–19% in rural Vietnamese non-pregnant women [17], [19], [27]. The residual anemia in this population does not appear to be due to deficiency of iron or other hematinic micronutrients (ie folate, B12, vitamin A) [19]. In the current study, we show that residual anemia prevalence is significantly higher in ethnic sub-populations. We believe that anemia related to an undiagnosed hemoglobinopathy is a likely explanation for the persistent anemia observed in women in Yen Bai, where more than 30% belong to ethnic minority groups known to have a higher rate of hemoglobin variants [28]. Importantly, our results show that residual anemia is not accompanied by iron deficiency after 54 months of program operation. Nor is there evidence of chronic iron overload in these populations.

This study has some limitations. As a population-based and long-term program, there were gaps in data collection, particularly with regard to the impact of the educational and promotional program on changing lifestyle habits. While not shown here, data suggests that women increasingly washed their hands after working and before preparing food. A missed opportunity was to identify pregnant women and record birth outcomes, thus recording any programmatic influence on change over time in mean birth weight and other maternal and infant outcomes of interest. To redress this, a sub-study comparing birth weights recorded in the initial trial districts and those recorded in two other districts of the province prior to the expansion of the intervention was carried out. The results showed statistically significant differences in low birth weight prevalence and mean birth weight between the two study arms [29].

Another limitation was the lack of a control group, which raises the possibility that external factors such as improved living standards, changed behaviours and improved nutrition may have accounted for some of the observed improvements in parasitological and haematological measures. However it should be recognized that, for ethical reasons, it would have been impossible to exclude a group of women infected with intestinal helminths from treatment for 5 years; and that evaluation of the long-term nutritional advantages would not have been possible over a shorter time-frame, Changes in economic circumstances were difficult to assess in Yen Bai, as the province does not have detailed year-on-year socio-economic data available. However, the global economic crisis occurred just after the study commenced and so it is likely that socioeconomic circumstances deteriorated, rather than improved, during this period. We did observe some basic infrastructure improvements during the 5 year study period, including upgrading of the main roads, but the immediate living standard of the rural population did not visibly change (Gerard Casey, personal communication).This is supported by the finding that protein intake, such as meat, egg and fish, did not change during the study; hand-washing behaviour did not show significant improvement; and there was no evidence of change in participants' employment status such as moving from basic farming work to paid employment. We did observe a decrease over time in the number of women who did not wear shoes while working, which may have influenced exposure to helminths infections.

The observation cohort was relatively small compared to the overall intervention population, which may have introduced bias. However, the cohort was randomly selected and indirect evidence suggested that it was broadly representative of the total population. In the sub-study by Passerini et al (2012) which did measure socio-economic indicators there was no difference between participants in different districts [29]. As well, the ethnic mix of our survey participants was similar to that of the province as a whole. (Luong Ba Phu, personal communication).

It has long been argued that sustainable long-term public health programs such as the control of vitamin and mineral deficiencies in a population require supportive government policy and adequate budget [30]. Unfortunately, these are also the most difficult steps to achieve for reasons that have not changed for decades. While the cost per individual may be minimal and the benefit-cost ratio high (the iron-folic acid and de-worming intervention in Yen Bai province has been assessed as having an annual cost of USD 0.76 per woman with a benefit-cost ratio of 6.7∶1) [31], the sheer weight of numbers ensures that, where policy makers do not take into account the societal and economic impact of iron deficiency, the cost per population appears excessive. For this reason, the recent donation of albendazole from GlaxoSmithKline and mebendazole from Johnson and Johnson (for a total of 600 million anthelminthics every year) is considered significant progress for the elimination of morbidity due to soil transmitted helminth infections in school age children and should, in our opinion, be extended to other risk groups such as pre-school children and women of child bearing age. As long as the discussion about benefits remains defined by the impact on maternal and infant health, it is likely that the timeframe for societal improvements will be perceived as too long to justify a national commitment.

To date, there have been few reports on long-term iron deficiency prevention interventions aimed at improving the general health of women in developing economies. One early intervention used iron-fortified sugar in 3 communities in semi-rural Guatemala. There were significant improvements of all iron nutrition indicators in most population groups [32]. Another was a large-scale effectiveness study in India that, similar to ours, was not planned as a long-term study nor did it have a control group. However, it was expanded due to popular grass roots demand. Weekly iron-folic acid supplementation distributed through community volunteers, combined with counseling, reduced the prevalence of anemia and improved hemoglobin levels in girls aged 11 to 18 years [33]. In the case of the Yen Bai intervention, program expansion was also encouraged by health authorities who acknowledged that it produced significant benefits for individual women and their communities. More recently reported is a one-year intervention that was undertaken among tribal people in northeastern states of India. Hemoglobin levels in the treatment arms improved following daily supplementation with iron-folic acid plus B12 or the supplement plus spirulina [34].

In 2004 it was explicitly acknowledged that “…iron deficiency has been seen as a ‘women's problem’ [35].” The discussion of women's nutrition, and specifically iron supplementation and fortification, is framed predominantly by the perception of women as reproductive and child nurturing units. Notable exceptions have been studies on the impact of iron supplementation on women's economic performance [9], [36]. Reports regarding the impact of iron supplementation on women's quality of life remain largely anecdotal and consequently unpublished. The importance of this health issue has been emphasized recently with the inclusion of guidelines and recommendations for intermittent iron-folic acid supplementation for women of reproductive age in WHO's e-Library of Evidence for Nutrition Actions (eLENA) [13], [14].

The clear lesson from both our study and that of Vir et al (2008) is that women in resource-poor settings, with little access to comprehensive primary health services or adequately nutritious diets, enthusiastically embrace nutrition initiatives and are willing to engage over the long term when regular community support is maintained. The WHO Regional Office for the Western Pacific has recently published a review of the effectiveness, cost and feasibility of weekly iron and folic acid deficiency for WRA, based on an analysis of 10 successful projects conducted in 6 countries, providing practical guidance for scaling up these programmes [37]. Following successful programmes targeting several million school-based and out-of-school adolescents, the Indian government recently established a national program of weekly iron and folic acid supplementation aimed at 120 million adolescents (Sheila Vir, personal communication) [38]. This intervention should be urgently considered in other settings with high rates of anemia in women of reproductive age, and where the health service extends to the village level, with strong support from local leaders.

## Supporting Information

Checklist S1 STROBE Checklist. STROBE statement: Checklist of items that should be included in reports of cohort studies.
(DOC)
